# Exploring the relationship between *GBA1* host genotype and gut microbiome in the *GBA1*^L444P/WT^ mouse model: implications for Parkinson’s disease pathogenesis

**DOI:** 10.3389/fnins.2025.1546203

**Published:** 2025-10-01

**Authors:** Elisa Menozzi, Mallia Geiger, Victoria Meslier, Federico Fierli, Marine Gilles, Kai-Yin Chau, Aymeric David, Revi Shahar Golan, Alexandre Famechon, Sofia Koletsi, Christian Morabito, Benoit Quinquis, Nicolas Pons, Stanislav Dusko Ehrlich, Jane Macnaughtan, Mathieu Almeida, Anthony HV Schapira

**Affiliations:** ^1^Department of Clinical and Movement Neurosciences, UCL Queen Square Institute of Neurology, London, United Kingdom; ^2^Aligning Science Across Parkinson’s (ASAP) Collaborative Research Network, Chevy Chase, MD, United States; ^3^Université Paris-Saclay, INRAE, MGP, Jouy-en-Josas, France; ^4^Liver Failure Group, Institute for Liver and Digestive Health, University College London, London, United Kingdom

**Keywords:** gut microbiome, microbiota-gut-brain axis, Parkinson disease, glucocerebrosidase, GBA

## Abstract

**Background:**

Heterozygous variants in *GBA1* are the commonest genetic risk factor for Parkinson’s disease (PD), but penetrance is incomplete. *GBA1* dysfunction can cause gastrointestinal disturbances and microbiome changes in preclinical models. Mounting evidence suggests that the microbiota–gut–brain axis is potentially implicated in PD pathogenesis. Whether the gut microbiome composition is influenced by host *GBA1* genetics in heterozygosis has never been explored.

**Objectives:**

This study aimed to evaluate whether heterozygosity for the *GBA1* pathogenic L444P variant can cause perturbations in gut microbiome composition.

**Methods:**

Faecal samples collected from *GBA1*^L444P/WT^ and *GBA1*^WT/WT^ mice at 3 and 6 months of age were analysed through shotgun metagenomic sequencing.

**Results:**

No differences in *α*- and *β*-diversities were detected between genotyped groups, at either time point. Overall, we found a little variation in the gut microbiome composition and functional potential between *GBA1*^L444P/WT^ and *GBA1*^WT/WT^ mice over time.

**Conclusion:**

Host *GBA1* genotype does not impact gut microbiome structure and composition in the presented *GBA1*^L444P/WT^ mouse model. Studies investigating the effect of a second hit on gut physiology and microbiome composition could explain the partial penetrance of *GBA1* variants in PD.

## Introduction

The aetiology of Parkinson’s disease (PD) is complex and multifactorial, resulting from both genetic and non-genetic factors (Tanner and Ostrem, 2024). Variants in the *GBA1* gene are found in approximately 10–15% of PD patients, thus representing the commonest genetic risk factor for PD (Petrucci et al., 2020; Skrahina et al., 2021). The *GBA1* gene encodes the lysosomal enzyme glucocerebrosidase (GCase), which breaks down glucosylceramide (GluCer) to ceramide and glucose. Biallelic pathogenic variants in the *GBA1* gene cause Gaucher disease, which is characterised by a deficiency in GCase enzymatic activity, resulting in the excessive accumulation of GluCer in multiple innate and adaptive immune cells in the spleen, liver, lung, and bone marrow (Pandey et al., 2017).

People with PD carrying *GBA1* variants, especially pathogenic severe variants such as p.L483P (more commonly known as L444P), display a more severe clinical phenotype, characterised by a higher burden of autonomic symptoms and olfactory dysfunction (Carandina et al., 2022; Menozzi and Schapira, 2021). Moreover, severe *GBA1* variants are associated with the highest risk of developing PD (odds ratio up to 30.4) (Vieira et al., 2024). Despite the high frequency of *GBA1* variants detected in PD cases, most individuals carrying homozygous or heterozygous variants in the *GBA1* gene will not develop PD over their lifetime (Hertz et al., 2024). The additional factors that can contribute to an increase in the risk of developing PD in *GBA1* variant carriers have not yet been identified (Menozzi et al., 2023).

Accumulating research points towards the gastrointestinal tract as a potential initial site of PD pathological changes. Preclinical evidence showed that the caudo-rostral spread of *α*-synuclein pathology from the gastrointestinal tract to the brain could occur after intestinal inoculation of *α*-synuclein pre-formed fibrils or α-synuclein derived from human PD brain lysate (Kim et al., 2019; Challis et al., 2020; Holmqvist et al., 2014). Moreover, postmortem and multi-modal imaging studies support the hypothesis that there is a subgroup of patients with PD who could manifest initial α-synuclein pathology in the gastrointestinal tract, namely in the enteric nervous system, with subsequent propagation to the dorsal motor nucleus of the vagus, sympathetic nervous system, brainstem, and the rest of the central nervous system (the so-called ‘body-first’ PD subtype) (Horsager et al., 2020; Borghammer et al., 2022). In contrast, the ‘brain-first’ subtype of PD is characterised by the initial deposition of *α*-synuclein pathology in the olfactory bulb and/or limbic system, with subsequent spread to the brainstem and peripheral nervous system (Horsager et al., 2020). Clinically, body-first and brain-first PD patients would differ, with the former presenting more severe autonomic and olfactory dysfunction compared to the latter (Horsager et al., 2022).

Considering the importance of the gastrointestinal tract in body-first PD pathogenesis, in recent years, increasing attention has been drawn to the potential contribution of the gut microbiome to this process. Exposure to gut microbial components such as lipopolysaccharide and lipopeptide enhanced intracellular levels of *α*-synuclein protein in a murine model of gut enteroendocrine cells (Hurley et al., 2023). Transplantation of faecal material from people with PD promoted motor symptoms and pathological changes in different mouse models, suggesting that alterations in the gut microbiome might trigger PD pathology (Sampson et al., 2016; Sun et al., 2018). Indeed, perturbations of the gut microbiome composition have extensively been reported in several populations of PD patients, without a specific genetic background (Nishiwaki et al., 2024). These perturbations seem to be particularly evident in body-first PD patients compared to brain-first PD (Park et al., 2024).

Because of the similarities in clinical profile displayed by PD patients with pathogenic *GBA1* variants and body-first PD patients, it has been recently hypothesised that *GBA1*-associated PD would more often fit within the body-first PD phenotype (Horsager et al., 2022). Hence, it is reasonable to interrogate the role of the gut microbiome in *GBA1*-associated PD. If and how pathogenic *GBA1* variants *per se* impact gastrointestinal function and microbiome composition and thus contribute to PD pathogenesis has been investigated in very few studies.

Animal models have shown that GCase deficiency can contribute to *α*-synuclein accumulation in the gastrointestinal tract (Challis et al., 2020). Moreover, heterozygosity for the severe pathogenic L444P *GBA1* variant reduced α-synuclein degradation, induced an earlier onset of pathological phosphorylated α-synuclein, and exacerbated both motor and gastrointestinal dysfunction in mice also carrying the mutated human *SNCA* A53T (hSNCA^A53T^/*GBA1*^L444P^) compared to hSNCA^A53T^ mice, wild-type for *GBA1* (Fishbein et al., 2014). Delayed intestinal transit time, decreased faecal pellet output, and compromised intestinal barrier integrity were detected in aged flies lacking the *Gba1b* gene, the main fly orthologue of *GBA1* (Atilano et al., 2023). In terms of gut microbiome composition, an increased bacterial load and an increased relative abundance of specific genera such as *Acetobacter* and *Lactobacillus* characterised *Gba1b^−/−^* flies; when raised under germ-free conditions, flies showed increased lifespan, improved locomotor abilities, and reduced glial activation (Atilano et al., 2023).

In this study, we aimed to explore for the first time the potential impact of mammalian host severe pathogenic variants in the *GBA1* gene (L444P) on the gut microbiome composition compared to wild-type controls.

## Methods

### Animals

Mice were treated in accordance with local ethical committee guidelines and the UK Animals (Scientific Procedures) Act 1986. All procedures were carried out in accordance with Home Office guidelines (United Kingdom; Project Licence Number: PP9638474). Male B6;129S4- Gbatm1Rlp/Mmnc (000117-UNC) mice expressing heterozygous knock-in L444P (also known as p.L483P) mutation in the murine *GBA1* gene (*GBA1*^L444P/WT^) were originally purchased from the Mutant Mouse Regional Resource Centre (MMRRC). Littermates (*GBA1*^WT/WT^) were used as controls. Within the first 21 days of life, mice were genotyped as previously described (Migdalska-Richards et al., 2016). After weaning on day 21, they were separated according to genotype into different cages and co-housed together for a maximum of 2 animals per cage. Faecal samples were collected for 3 consecutive days. On each day of collection, mice were removed from their home cages and singly housed for a maximum of 30 min, with water and diet provided during this time. After 30 min, faecal samples were collected avoiding urine contamination and immediately placed in airtight Eppendorf tubes into dry ice, until transferred to the laboratory where samples were stored at −80°C prior to further analyses. Faecal samples were collected at 3 and 6 months of age. Faecal pellet output was recorded on each day of collection, and the total faecal output was calculated at the end of collection by summing the weights of faecal pellets collected on each day. The animals’ weight was recorded at each collection time point.

### Metagenomics

DNA extraction, high-throughput sequencing, read mapping, and bioinformatical analysis to determine Metagenomic Species Pangenome (MSP) were performed for the study of the mice microbiota (DOI: dx.doi.org/10.17504/protocols.io.bp2l6x5wklqe/v1).

#### DNA extraction and high-throughput sequencing

Frozen faecal materials were aliquoted to ≤250 mg, and DNA extraction was performed following the procedure previously described in dx.doi.org/10.17504/protocols.io.dm6gpjm11gzp/v1, with the following modifications. 250 μL of guanidinium thiocyanate, 40 μL of N-lauroyl sarcosine (10% solution), and 500 μL of N-lauroyl sarcosine (5% solution in PBS 1X) were added to each frozen mouse faecal sample, which was then homogenised with a toothpick, vortexed, and transferred to a deep-well plate containing 400 μL of 0.1 mm glass beads (not in suspension). Subsequently, the sample plate was incubated at 70°C in a thermomixer for 1 h, with stirring at 1,400 rpm. Following centrifugation of the plate at 3,486 × g for a period of 5 min, the lysate was collected in a new plate. The pellet was then washed with 500 μL of TENP (50 mM Tris–HCL 20 mM EDTA 10 mM NaCl, saturated with PVPP). The plate was vortexed and centrifuged at 3,486 × g for 5 min, after which the recovered lysate was pooled with the previous one. Finally, the final lysate was centrifuged for 10 min at 3,486 × g, after which 800 μL were collected in a new plate. This plate was employed for purification with magnetic beads on the QIASymphony. The utilised protocol has been designed for MGP with the QIAGEN DSP Virus/Pathogen kit. DNA was quantified using Qubit Fluorometric Quantitation (Thermo Fisher Scientific, Waltham, US) and qualified using DNA size profiling on a Fragment Analyzer (Agilent Technologies, Santa Clara, US). One μg of high molecular weight DNA (>10 kbp) was used to build the library. Shearing of DNA into fragments of approximately 150 bp was performed using an ultrasonicator (Covaris, Woburn, US), and DNA fragment library construction was performed using the Ion Plus Fragment Library and Ion Xpress Barcode Adapters Kits (Thermo Fisher Scientific, Waltham, US). Purified and amplified DNA fragment libraries were sequenced using the Ion Proton Sequencer (Thermo Fisher Scientific, Waltham, US), with a minimum of 20 million high-quality 150 bp reads generated per library (Meslier et al., 2022).

#### Read mapping

Reads were quality-filtered to remove any low-quality sequences using AlienTrimmer software (Criscuolo and Brisse, 2013) and potential host-related reads using Bowtie2 (Langmead and Salzberg, 2012). Resulting high-quality reads were mapped onto the 5 million gene integrated reference catalogue of the Murine Intestinal Microbiota Integrated Catalog v2 (MIMIC2) (Plaza Onate et al., 2021) using the METEOR software suite (Pons et al., 2010). Read mapping was performed in a two-step procedure, using an identity threshold of 95% to the reference gene catalogue with Bowtie2 (Langmead and Salzberg, 2012). First, unique mapped reads were attributed to their corresponding genes. Second, shared reads were weighted according to the ratio of unique mapping counts. A downsizing procedure was performed to normalise the gene counts between samples by randomly selecting a subset of reads depending on the sequencing depth (usually ≥10 M reads for an average of 20 M reads depth sequencing). The gene abundance table was then normalised using the FPKM strategy and analysed using MetaOMineR (momr) R package^1^ (Le Chatelier and Prifti, 2023).

#### MSP microbial species determination

MSPs were used to quantify species associated with the 5.0 million gene MIMIC2 reference catalogue (Nielsen et al., 2014; Plaza Onate et al., 2021). MSPs are clusters of co-abundant genes (min size ≥500 genes) that likely belong to the same microbial species, reconstructed from the 5 million genes catalogue into 1,252 MSPs. MSP abundance profiles were calculated as the mean abundance of 100 marker genes, defined as the robust centroids of each MSP cluster. A threshold of 10% of the marker genes was applied as the MSP detection limit. Taxonomical annotation was performed using GDTB R07-RS207 (Parks et al., 2022).

#### Assessment of microbial functional potentials

To determine the functional potential of the gut microbiota at the module level, we used an INRAE pipeline as previously described (Thirion et al., 2023). Three databases were used to predict gene functions: Kyoto Encyclopedia of Genes and Genomes (KEGG) (Kanehisa and Goto, 2000), eggNOG database (version 3.0) (Huerta-Cepas et al., 2016), and TIGRFAMs (version 15.0) (Haft et al., 2001). First, genes of the 5 million genes catalogue were annotated using the KEGG107 database using Diamond (Buchfink et al., 2015) and further clustered into functional pathway modules according to KEGG Orthology (KO) groups, gut metabolic modules (GMMs) (Vieira-Silva et al., 2016), and gut–brain modules (GBMs) (Valles-Colomer et al., 2019). Second, KEGG, GMMs, and GBMs were reconstructed in each MSP using their reaction pathways based on their detected annotated KO, NOGs, or TIGRFAM genes. GMMs and GBMs were selected because they are specific to gut bacterial and gut–brain axis functions. For each pair of MSP/mouse, the completeness of any given functional module was calculated by considering the set of genes detected in the MSP of each mouse and the MSP completeness in each mouse. For a given MSP in a specific mouse, the completeness of the modules was corrected by the abundance of the MSP. After correction, functional modules in each MSP/mouse were considered complete if at least 90% of the involved reactions were detected. The abundance of functional modules in each sample was computed as the sum of the MSP abundances containing the complete functional module.

### Statistical analysis

Statistical analysis and visualisation were performed with R software (version 4.4.1) (R Core Team, 2019).

Group differences (WT/WT vs. L444P/WT) in faecal pellet output, animal weights, richness, *α*-diversity measures, and contrasts in species and functional module abundances were computed using the non-parametric Wilcoxon rank-sum test. For comparisons over time, the Wilcoxon signed-rank test was applied. The effect size was calculated as Cliff’s Delta. Differences in the prevalence of each species in each group, defined as the proportion of samples containing that species in relation to the total number of samples in the respective group, were calculated using Fisher’s exact test. Shannon diversity index was computed based on the MSP matrix using the function *diversity* from the R package vegan (Dixon, 2003). Principal coordinates analysis (PCoA) was performed using the R package ade4 (Thioulouse et al., 2018) on the Bray-Curtis dissimilarity index, which was computed with the vegdist function from the vegan package (Oksanen et al., 2012). Additionally, PCoA was performed on the Weighted UniFrac distance, which was computed using the phyloseq (McMurdie and Holmes, 2013) and ape (Paradis et al., 2004) R packages. First, the phylogenetic tree was loaded and filtered to retain only the taxa present in the samples using the *read.tree* and *keep.tip* functions from the ape package. Then, a phyloseq object was created by combining the phylogenetic tree, MSP matrix, and metadata using the phyloseq function from the phyloseq package. Finally, the Weighted UniFrac distance was calculated with the UniFrac function from the phyloseq package. Permutational multivariate analysis of variance (PERMANOVA) was computed using distance matrices with the function *adonis2* from the R package vegan (*n* = 1000 permutations), to assess differences in *β*‐diversity (Dixon, 2003).

The data, code, protocols, and key lab materials used and generated in this study are presented in a Key Resource Table alongside their persistent identifiers (Table 1) and available at https://doi.org/10.5281/zenodo.14864459.

**Table 1:** Key Resource Table summarising the data, code, protocols, and laboratory materials used and generated in this study.

**Resource Type**	**Resource Name**	**Source**	**Identifier**	**New/reuse**	**Additional Information**
Dataset	Metagenomics sequencing data	ENA (European Nucleotide Archive)	https://www.ebi.ac.uk/ena/browser/view/PRJEB86012	new	
Dataset	MSP abundance table	Zenodo	https://zenodo.org/records/15757420	new	
Dataset	Read alignment	Zenodo	https://zenodo.org/records/15756828	new	
Dataset	MIMIC2 reference catalogue	MetaGenoPolis, INRAE (National Research Institute for Agriculture, Food, and Environment)	https://entrepot.recherche.data.gouv.fr/dataset.xhtml?persistentId=doi:10.15454/L11MXM	reuse	
Dataset	GDTB R07-RS207	Sourmash project	https://sourmash.readthedocs.io/en/latest/databases.html	reuse	
Dataset	KEGG (version 107)	Kanehisa Laboratories	https://www.kegg.jp/kegg/download/%20(RRID:SCR_001120)	reuse	
Dataset	eggNOG (version 3.0)	EMBL (European Molecular Biology Laboratory)	http://eggnog5.embl.de/#/app/home%20(RRID:SCR_002456)	reuse	
Dataset	TIGRFAMs (version 15.0)	J. Craig Venter Institute (JCVI)	https://tigrfams.jcvi.org/cgi-bin/Terms.cgi%20(RRID:SCR_005493)	reuse	
Protocol	Protocol for DNA extraction, high-throughput sequencing, read mapping, and bioinformatical analysis to determine Metagenomic Species Pangenome (MSP) for the study of the mice microbiota	protocols.io	dx.doi.org/10.17504/protocols.io.bp2l6x5wklqe/v1	new	
Software/code	Code	Zenodo	https://zenodo.org/records/15757420	new	
Software/code	AlienTrimmer (version 0.4.0)	Institut Pasteur	https://gitlab.pasteur.fr/GIPHy/AlienTrimmer%20(RRID:SCR_011835)	reuse	
Software/code	Bowtie2 (version 2.5.4)	John Hopkins University	https://bowtie-bio.sourceforge.net/bowtie2/index.shtml%20(RRID:SCR_016368)	reuse	
Software/code	Meteor legacy (version 3.2.1)	MetaGenoPolis, INRAE (National Research Institute for Agriculture, Food, and Environment)	https://www.biorxiv.org/content/10.1101/2024.12.15.627490v1.full.pdf+html	reuse	
Software/code	R (version 4.4.1)	R project	https://www.r-project.org/%20(RRID:SCR_001905)	reuse	
Software/code	Diamond (version 2.1.9)	Diamond Github repository	https://github.com/bbuchfink/diamond%20(RRID:SCR_009457)	reuse	
Software/code	vegan (version 2.6.6.1)	CRAN	https://cran.r-project.org/web/packages/vegan/index.html%20(RRID:SCR_011950)	reuse	
Software/code	ade4 (version 1.7.22)	CRAN	https://cran.r-project.org/web/packages/ade4/index.html%20(RRID:SCR_024259)	reuse	
Software/code	phyloseq (version 1.48.0)	Phyloseq Github repository	https://joey711.github.io/phyloseq/%20(RRID:SCR_013080)	reuse	
Software/code	ape (version 5.8)	R package	https://cran.r-project.org/web/packages/ape/index.html%20(RRID:SCR_017343)	reuse	
Software/code	momr (version 1.31)	Momr Github repository	http://github.com/eprifti/momr	reuse	
Software/code	ggplot2 (version 3.5.1)	CRAN	https://cran.r-project.org/web/packages/ggplot2/index.html%20(RRID:SCR_014601)	reuse	
Software/code	ggpubr version (0.6.0)	CRAN	https://cran.r-project.org/web/packages/ggpubr/index.html%20(RRID:SCR_021139)	reuse	
Software/code	ggh4x (version 0.2.8)	CRAN	https://cran.r-project.org/web/packages/ggh4x/index.html	reuse	
Software/code	patchwork (version 1.2.0)	CRAN	https://cran.r-project.org/web/packages/patchwork/index.html%20(RRID:SCR_000072)	reuse	
Software/code	dplyr (version 1.1.4)	CRAN	https://cran.r-project.org/web/packages/dplyr/index.html%20(RRID:SCR_016708)	reuse	
Software/code	tidyverse (version 2.0.0)	CRAN	https://cran.r-project.org/web/packages/tidyverse/index.html%20(RRID:SCR_019186)	reuse	
Software/code	kableExtra (version 1.4.0)	CRAN	https://cran.r-project.org/web/packages/kableExtra/vignettes/awesome_table_in_html.html	reuse	
Software/code	stats (version 4.4.1)	Ecole Polythechnique fédérale de Zurich	https://stat.ethz.ch/R-manual/R-devel/library/stats/html/00Index.html%20(RRID:SCR_025968)	reuse	
Experimental model: Organism/ strain	GBA1 L444P/WT	B6;129S4-Gba1tm1Rlp/Mmnc (strain name); Mutant Mouse Resource & Research Centers (vendor); 000117-UNC (catalog number); RRID:MMRRC_000117-UNC (RRID)	https://www.mmrrc.org/catalog/sds.php?mmrrc_id=117	reuse	
Experimental model: Organism/ strain	GBA1 WT/WT	B6;129S4-Gba1tm1Rlp/Mmnc (strain name); Mutant Mouse Resource & Research Centers (vendor); 000117-UNC (catalog number); RRID:MMRRC_000117-UNC (RRID) - The control mice were the littermate controls generated from the same mouse line as the mutant animals	https://www.mmrrc.org/catalog/sds.php?mmrrc_id=117	reuse	

## Results

### Study cohort

An overview of the characteristics of the study cohort is presented in Table 2. A total of 16 WT/WT and 13 L444P/WT animals were included in the study (with 1 L444P/WT sampled only at 3 months). No difference in the total faecal output weight was detected at 3 or 6 months between genotypes or between time points within each group. We observed a significant weight gain between 3 and 6 months of age within the WT/WT group (*p* = 0.000031) and within the L444P/WT group (*p* = 0.00049). At 6 months, the median weight of the L444P/WT group was significantly higher than the one in the WT/WT group (*p* = 0.017), but the difference between 3 and 6 months (delta weight) only showed a trend towards significance, being higher in L444P/WT (*p* = 0.053).

**Table 2:** Overview of study cohort.

Genotype	WT/WT		L444P/WT		*p* value	
Age (months)	3	6	3	6	P1	P2
*N*	16	16	13	12		
Faecal output (g)	0.72	0.67	0.62	0.78	ns	ns
Animal weight (g)	31.50	36.67	33.35	42.06	ns	0.017

Data are presented as median values. P1 = comparison between WT/WT and L444P/WT at 3 months; P2 = comparison between WT/WT and L444P/WT at 6 months. *p* values, as determined by Wilcoxon rank-sum test, are reported in the table (ns = not significant).

### Limited variation of the gut microbiome structure between groups

**Figure 1:**
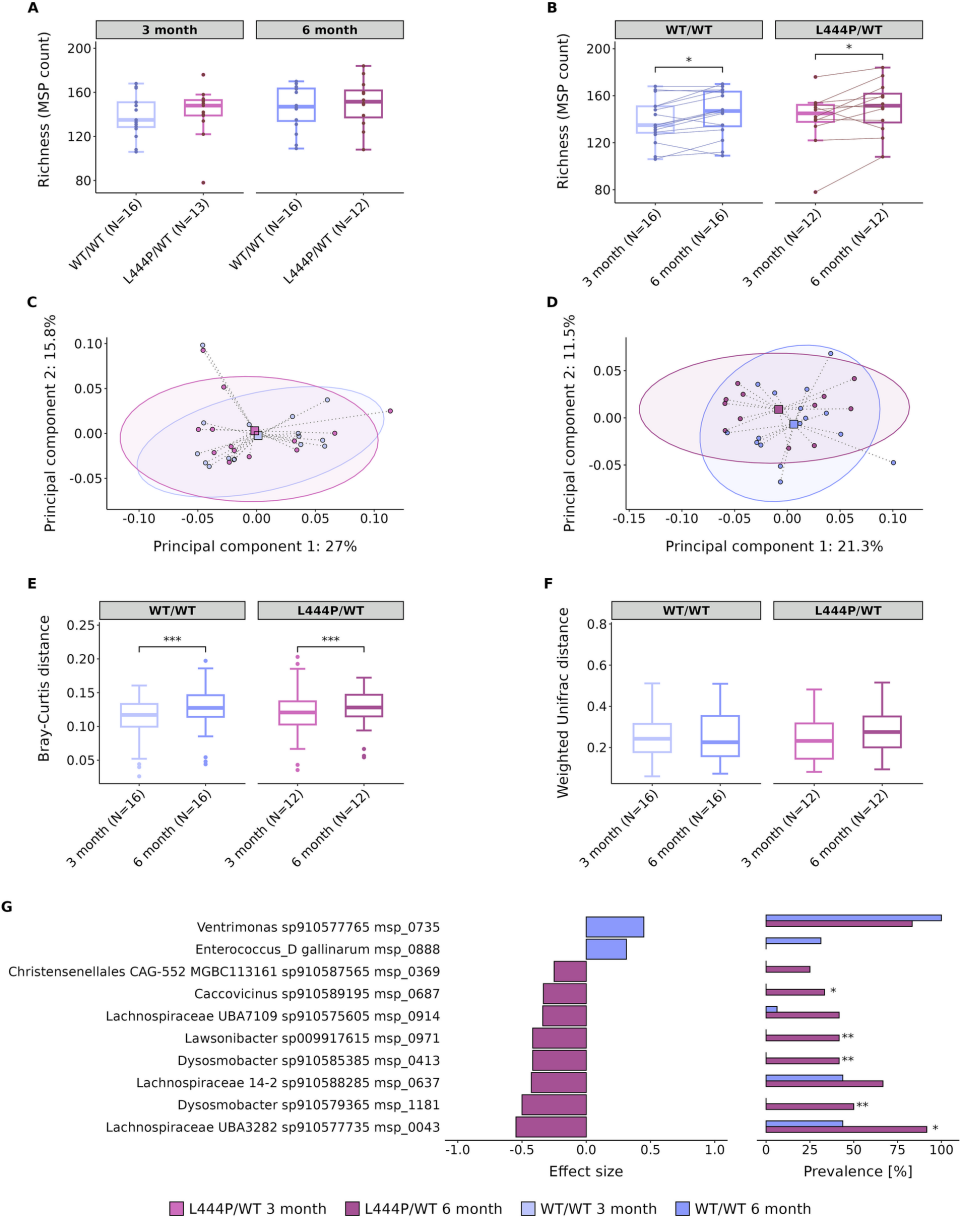
Gut microbiome alterations in *GBA1*^L444P/WT^ and *GBA1*^WT/WT^ mice. **(A)** Differences in species (MSP) richness between groups (WT/WT and L444P/WT) at 3 months and 6 months. **(B)** Differences in species (MSP) richness within the WT/WT group and the L444P/WT group over time. PCoA performed on the Bray–Curtis dissimilarity index at 3 months **(C)** and 6 months **(D)**. **(E)** Differences in the Bray–Curtis dissimilarity index between the WT/WT and L444P/WT groups at different time points. **(F)** Weighted UniFrac distance metric within the WT/WT and L444P/WT groups at different time points. **(G)** Microbial species abundance between WT/WT and L444P/WT animals at 6 months: left, diverging bar plots show the relative abundance of species in WT/WT and L444P/WT according to the effect size measured by the Cliff’s Delta; right, bar charts show the percentage of prevalence of species between groups. Significant differences, determined using Wilcoxon’s signed-rank test and Fisher’s exact test, are indicated by a star above each box plot and bar plot (* = *p* < 0.05, ** = *p* < 0.01, and *** = *p* < 0.001).

Gene count and MSP richness were not found to be significantly different between groups at different time points (*p* = 0.33 and *p* = 0.55 for MSP richness, at 3 and 6 months, respectively, Figure 1A). This finding was further confirmed by analysing additional *α*-diversity indices, including the Shannon index. The Shannon index was 3.61 and 3.67 for the WT/WT and L444P/WT groups at 3 months (*p* = 0.47) and 3.61 and 3.71 at 6 months (*p* = 0.66), respectively. In line with weight, MSP richness was found to increase over time in each group (*p* = 0.012 and *p* = 0.034 for MSP richness in the WT/WT and L444P/WT groups, respectively, Figure 1B).

We also performed β-diversity analysis as measured by the Bray–Curtis dissimilarity index. We did not observe a significant difference between genotypes at either of the time points (*p* = 0.73 at 3 months and *p* = 0.52 at 6 months, Figures 1C,D, respectively), although a significant variation in the structure of the gut microbiome between 3 and 6 months of age was identified in both groups (*p* = 0.0009 for the WT/WT and *p* = 0.0009 for the L444P/WT, Figure 1E). Furthermore, when calculating the weighted UniFrac distance, we observed no significant differences between 3 and 6 months of age in both groups (*p* = 0.23 for the WT/WT and *p* = 0.29 for the L444P/WT, Figure 1F), suggesting that species changing over time within each group are phylogenetically closely related.

### Differentially abundant species and functional modules between groups

At 3 months, only one species was differentially abundant between groups, being enriched in the L444P/WT group (*Schaedlerella sp910575475*), confirming the little variation in the gut microbiome observed at 3 months of age. At 6 months, there were 10 species differentially abundant, with the majority of them only detected in one group (Figure 1G). Two species, detected in both groups but more abundant in the L444P/WT, belong to the family *Lachnospiraceae*, phylum Firmicutes_A. At 6 months, the proportion of enriched species in the L444P/WT group was higher than in the WT/WT group (Fisher’s exact test: *p* = 0.02).

In line with the limited differences observed for the microbial species, little variation was found in the functional potential between groups at each time point. Only one KEGG module enriched in WT/WT (ascorbate degradation, ascorbate = > D-xylulose-5P, M00550) and one GMM enriched in L444P/WT (glycocholate degradation, MF0044) were found to be differentially abundant between groups at 3 months. At 6 months, the same GMM enriched at 3 months in L444P/WT (MF0044) was also enriched, whereas no differences in KEGG or GBM were found between genotypes.

## Discussion

To the best of our knowledge, this is the first study to investigate the gut microbiome composition of mice carrying *GBA1* variants in heterozygosis, the most frequent genetic risk factor for PD. Our data did not show any significant perturbations of the gut microbiome structure and abundance of species or functional modules due to standalone *GBA1* genetic status. We found that the relative abundance of a few species was significantly different between groups at 6 months; however, most of these species were detected only in one group, thus making it difficult to draw any conclusions regarding a host genotype–microbiome interaction. The only species that were detected in both L444P/WT and WT/WT groups and were found to be more abundant in the former belonged to the Lachnospiraceae family. Species belonging to this family have been linked to anti-inflammatory or neuroprotective effects and reported to be under-represented in human PD cohorts compared to controls (Huang et al., 2023; Lubomski et al., 2022).

These findings are in line with the available literature on Gaucher disease. Gastrointestinal involvement is very rarely reported in Gaucher disease and, when present, is largely unresponsive to enzyme replacement therapy (Kim et al., 2017). Perturbations of the gut microbiome leading to fat malabsorption and intestinal dysfunction have been described in knockout mice for the *SCARB2* gene, which encodes the lysosomal integral membrane protein-2 (LIMP-2) which is fundamental to traffic GCase to the lysosome (Li et al., 2024); however, no report on gut microbiome in patients with Gaucher disease is currently available.

*GBA1* variant carriers represent a group of susceptible individuals at risk of future development of PD, but the limited penetrance of *GBA1* variants for PD implies that additional factors are needed for clinically manifesting disease expression. It has been suggested that in susceptible individuals, exposure to ingested toxicants or intestinal infections could result in low-grade gut inflammation and microbiome alterations, which could instigate *α*-synuclein pathology in the gut with subsequent rostral spread to the brain (Houser and Tansey, 2017; Dorsey et al., 2024; Khare et al., 2019; Wang et al., 2021; Ilieva et al., 2022; Zeng et al., 2022). Previous models of mild chronic colitis using dextran sodium sulphate (DSS) induced more severe motor dysfunction, microglia activation, and dopaminergic neuron loss in genetic models of PD such as mice carrying the *LRRK2* G2019S variant (Lin et al., 2022). Recent studies investigating the effect of DSS or paraquat, a neurotoxic herbicide, found that these stimuli differentially interacted with specific genotypes including the hSNCA^A53T^/hSNCA^A53T^ or the hSNCA^A53T^/*GBA1*^L444P^, leading to toxicant-genotype-specific microbiome alterations, gastrointestinal dysfunction, and neurodegenerative changes (Chaklai et al., 2024). We could therefore hypothesise that the combination of *GBA1* host genetics and exposure to ingested toxicants and/or gastrointestinal inflammation (dual-hit hypothesis) could represent a possible pathogenetic mechanism underlying *GBA1*-PD development which is mediated by gut microbiome perturbations and gastrointestinal dysfunction.

Indeed, as mentioned above, increasing evidence suggests that disruption of gut microbiome homeostasis might cause the accumulation of pathological *α*-synuclein in the GI tract and, in particular, in the enteric nervous system, one of the assumptions of the ‘body-first’ hypothesis of PD (Anis et al., 2023). However, the precise mechanisms linking gut dysbiosis to *α*-synuclein accumulation are still not fully understood. For instance, the Gram-negative bacterial endotoxin lipopolysaccharide (LPS), which is recognised by Toll-like receptor 4 (TLR4), has been shown to play a role in PD pathogenesis at the GI level, possibly through mediation of the inflammatory processes (Anis et al., 2023). The systemic exposure of mice to low doses of LPS induced increased intestinal *α*-synuclein expression and permeability, and pathological α-synuclein accumulation in myenteric neurons (Kelly et al., 2014). Increased expression of TLR4 mRNA was found in intestinal mucosal biopsies from PD patients compared to controls, alongside an increase in pro-inflammatory cytokine and chemokine expression and intestinal barrier disruption markers (Perez-Pardo et al., 2019). The colonisation of α-synuclein-overexpressing mice with curli-producing *Escherichia coli* exacerbated motor and GI impairment and promoted α-synuclein aggregation and inflammation in both the gut and the brain; however, these data did not determine which of these processes (α-synuclein aggregation or inflammation) was the primary driver of curli-mediated pathophysiology (Sampson et al., 2020). In one of our previous studies, we showed that exposure to LPS or lipopeptides, the latter recognised by TLR2, increased α-synuclein protein expression and release in gut enteroendocrine cells, without any significant changes in α-synuclein mRNA expression or alterations in the ubiquitin–proteasome system, and induced pro-inflammatory responses (Hurley et al., 2023). In our model, specific antagonists to TLR4 and TLR2 prevented α-synuclein protein changes in response to LPS or lipopeptides, suggesting a direct role of these stimuli (Hurley et al., 2023). Overall, the above results seem to suggest that neuroinflammation could be an important mechanism linking microbiome alterations to pathological α-synuclein aggregation; however, other mechanisms including impairment in α-synuclein clearance (e.g., macroautophagy and chaperone-mediated autophagy) might also be implicated in favouring and/or aggravating *α*-synuclein accumulation. This might be particularly true in the context of *GBA1*-associated PD where the impairment in the autophagy lysosomal pathway or chaperone-mediated autophagy secondary to GCase dysfunction has been repeatedly found to contribute to α-synuclein accumulation (Chatterjee and Krainc, 2023; Kuo et al., 2022). Interestingly, following *GBA1* gene transfer in α-synuclein-overexpressing mice, intestinal α-synuclein pathology and enteric network connectivity were rescued, suggesting the potential effect of GCase dysfunction on gut physiology and the use of peripheral *GBA1* as a therapeutic target (Challis et al., 2020).

We acknowledge that our study has some limitations. First, mice were sacrificed at 6 months of age, so potential changes in the gut microbiome consequent to ageing might have been missed, although in our pilot study, we did not observe any dramatic difference in gut microbiome composition between 6 months and later time points (data shown in Supplementary material). The potential contribution of age-associated microbiome changes to the immune system homeostasis and the mucosal barrier permeability has generated interest in recent years, and future studies should address the complex relationship between microbial dysbiosis, immune senescence, and inflammaging (Conway and Duggal, 2021). Second, we used only male mice to eliminate sex, a factor driving differences in the gut microbiome composition (Valeri and Endres, 2021). By doing this, we might have missed any sex-specific interactions between the *GBA1* host genotype, gut microbiome, and the immune system that might play a role in PD pathogenesis, as recently shown in other neurodegenerative conditions (Bostick et al., 2024). Third, we did not perform any measurements of gastrointestinal transit time, inflammation, or permeability, as our primary focus was to evaluate host genotype–microbiome interactions. Considering the high frequency of GI disturbances in people with PD, and in particular in body-first PD patients (Horsager et al., 2022), it is important to design future studies including such measurements to fully evaluate the impact of host *GBA1* genotype on gut physiology. Fourth, we did not analyse levels of α-synuclein, neuroinflammatory markers, or GCase activity in the gut mucosa. These analyses, alongside the use of more complex genetic models such as the hSNCA^A53T^/*GBA1*^L444P^ model, might also help better clarify the role of *GBA1* in PD pathogenesis.

Notwithstanding, the design of our study (different genotypes kept in separate cages within the same biological facility and under the same dietary conditions) minimised the risk of gut microbiome homogenisation between different genotyped animals due to coprophagia, as previously observed (Supplementary Figure 1). Thus, our study represents an essential preliminary step to evaluate the validity of the dual-hit hypothesis for *GBA1*-PD pathogenesis in future models.

## Data Availability

The data, code, protocols, and key lab materials used and generated in this study are listed in a Key Resource Table alongside their persistent identifiers at https://doi.org/10.5281/zenodo.14864459.

## Footnotes

^1^ https://cran.r-project.org/web/packages/momr/index.html
